# Recombinant production of influenza hemagglutinin and HIV-1 GP120 antigenic peptides using a cleavable self-aggregating tag

**DOI:** 10.1038/srep35430

**Published:** 2016-11-03

**Authors:** Wanghui Xu, Qing Zhao, Lei Xing, Zhanglin Lin

**Affiliations:** 1 Department of Chemical Engineering, Tsinghua University, One Tsinghua Garden Road, Beijing 100084, China

## Abstract

The increasing demand for antigenic peptides in the development of novel serologic diagnostics and epitope-based vaccines requires rapid and reliable peptide synthesis techniques. Here we investigated a method for efficient recombinant expression and purification of medium- to large-sized antigenic peptides in *E. coli*. Previously we devised a streamlined protein expression and purification scheme based on a cleavable self-aggregating tag (cSAT), which comprised an intein molecule and a self-aggregating peptide ELK16. In this scheme, the target proteins were fused in the C-termini with cSAT and expressed as insoluble aggregates. After intein self-cleavage, target proteins were released into the soluble fraction with high yield and reasonable purity. We demonstrated the applicability of this scheme by preparing seven model viral peptides, with lengths ranging from 32 aa to 72 aa. By adding an N-terminal thioredoxin tag, we enhanced the yield of target peptides released from the aggregates. The purified viral peptides demonstrated high antigenic activities in ELISA and were successfully applied to dissecting the antigenic regions of influenza hemagglutinin. The cSAT scheme described here allows for the rapid and low-cost preparation of multiple antigenic peptides for immunological screening of a broad range of viral antigens.

Discrete peptides derived from intact viral antigens are important tools for immunological studies, including epitope mapping, peptide arrays and the characterization of protein–protein interactions^1^,^2^,^3^,^4^. From such studies, a number of antigenic peptides have been developed into novel serological diagnostics and epitope-based vaccines^5^,^6^,^7^,^8^. In particular, peptides over 40 amino acids (aa) have become increasingly important in vaccine development, mainly because they are better at forming stable structural domains and mimicking natural structural epitopes^6^. The increasing demand for medium- to large-sized peptides (30–100 aa) in immunological research has stressed the need for rapid and reliable peptide synthesis techniques, using or chemical or recombinant technologies. Despite progress in solid-phase peptide synthesis, the purification and recovery of chemical synthetic peptides of over 30 aa in length remains costly and success rates are largely sequence-dependent^9^. Recombinant peptides shorter than 100 aa are usually prone to proteolysis and difficult to express with high yield in commonly used microbial hosts, such as *E. coli* cells^10^,^11^,^12^. Here we investigated methods for the efficient recombinant expression and purification of antigenic peptides with lengths ranging from 30–100 aa in *E. coli* cells.

Fusion partners have been widely used to enhance the stability and solubility of recombinant proteins in *E. coli*^11^,^13^,^14^. To produce recombinant peptides with high yields, several emerging schemes employ fusion tags that induce the formation of protein inclusion bodies (IBs)^15^, including the well-known hydrophobic bacterial ketosteroid isomerase (KSI) applied in the commercial pET-31b(+) vector^16^, the autoprotease N^pro^ of classical swine fever virus^17^, the elastin-like polypeptide (ELP) tag^18^,^19^ and the recently reported N-terminal region (1–62 aa) of synthetic human growth hormone (GH)^20^. IBs can provide resistance to proteolytic effects, high expression rates, and simple recovery by centrifugation. However, the fusion tags described above are usually large in size (over 120 aa), increasing the burden of recombinant expression. Furthermore, separation of the target peptide from the tag usually involves harsh chemical cleavage (as for the KSI scheme), a tedious refolding procedure (as for the N^pro^ scheme), multiple phase transition cycling steps (as for the ELP scheme) or solubilization of the IBs using alkali solutions (as for the Cry4a scheme).

Previously, we reported that a short β-structured self-complementary peptide ELK16 (LELELKLKLELELKLK), when fused to a target enzyme in the C terminus, can induce the formation of highly active enzyme aggregates^21^. Based on this finding, we devised a convenient and matrix-free approach to express and purify recombinant proteins from *E. coli*, referred to as the cleavable self-aggregating tag (cSAT) scheme^22^. Briefly, the target proteins were fused at the N-terminal of the *Mxe* GyrA intein molecule followed by ELK16, expressed as insoluble aggregates and isolated by centrifugation. After dithiothreitol (DTT)-induced intein-mediated cleavage, the target proteins were released into the soluble fraction with high purity and yield. Here we further improved the cSAT scheme by adding an N-terminal thioredoxin (Trx) tag and demonstrated the usefulness of this scheme in efficiently expressing and purifying medium to large-sized viral antigenic peptides for immunological assays. Seven virus-derived peptides with lengths ranging from 32 to 72 aa were studied. Among them, five were derived from the pandemic 2009 A(H1N1) influenza virus hemagglutinin (HA) and two from the type 1 human immunodeficiency virus (HIV-1) envelope glycoprotein gp120^23^,^24^. All of the peptides were screened from random peptide libraries of viral surface antigens. In this study, the peptides both with and without the N-terminal Trx tag were expressed and purified from *E. coli* cells using the cSAT scheme. Trx was found to greatly enhance the yield and purity of soluble target peptides released from the aggregates. The purified viral peptides demonstrated high antigenic activities in ELISA and were successfully applied to dissect the important antigenic regions of influenza hemagglutinin^24^.

## Results

### Constructions of fusion proteins

**Figure 1:**
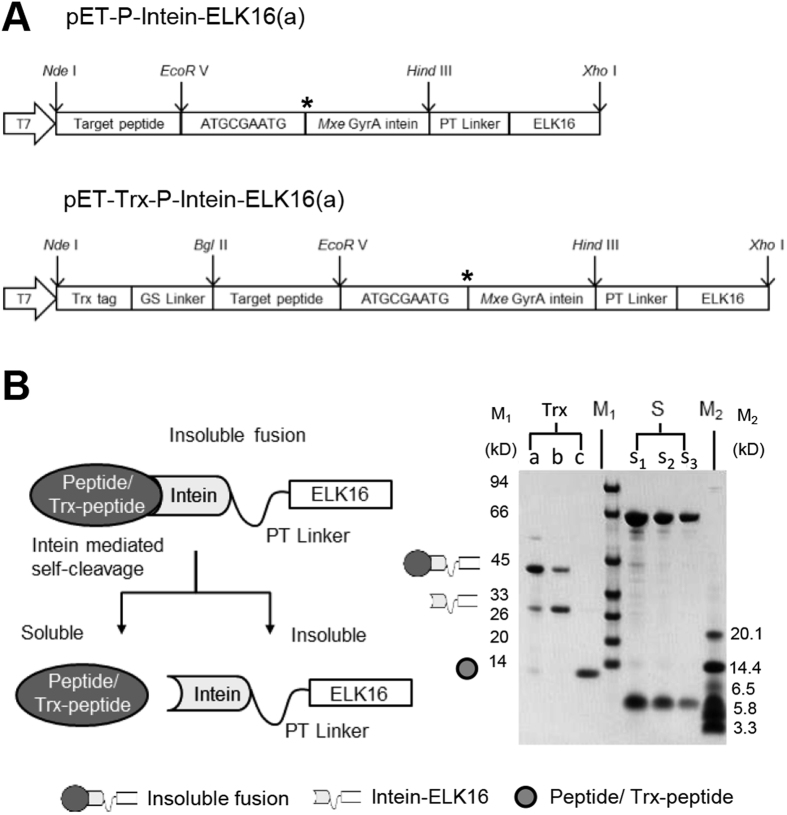
Construction of expression vectors for fusion proteins. (**A**) Expression vector pET-P-Intein-ELK16(a) for the fusion of target peptide-intein-ELK16 was based on the previously constructed vector pET30a-LipA-I-ELK16^22^ with the *EcoRI* site replaced with an *EcoRV* site. Expression vector pET-Trx-P-Intein-ELK16(a) for the fusion of Trx-target peptide-intein-ELK16 was then modified by inserting the thioredoxin (Trx) gene with a GS linker sequence between *NdeI* and *EcoRV* sites. The short segment ATGCGAATG encoding MRM was inserted to facilitate intein-mediated cleavage. ‘*’ indicates the intein cleavage site. (**B**) Schematic of the expression and purification of target peptide or Trx-target peptide, demonstrated by the SDS-PAGE results of Trx. Lane a, insoluble fraction of the cell lysate; lanes b and c, insoluble and soluble fractions of the cleaved fusion protein respectively; lanes s1, s2 and s3, quantification standards consisting of bovine serum albumin (BSA, 66.5 kDa) at 3, 1.5 and 0.75 μg/lane and aprotinin (6.5 kDa) at 1.5, 0.75 and 0.3 μg/lane respectively. The molecular weights of the protein standards M1 and M2 are indicated on the left and right sides respectively. The gel was cropped and the full-length gel is presented in Supplementary Fig. S3.

The fusion proteins were constructed using two sets of vectors in this study, as shown in Fig. 1A. The first vector pET-P-Intein-ELK16(a) was modified from a previously constructed vector pET30a-LipA-I-ELK16 and used to express the fusion of target peptide-intein-ELK16^22^. The other vector pET-Trx-P-Intein-ELK16(a) contained a thioredoxin tag (Trx), with a molecular weight of 12.5 kD, inserted upstream of the target peptide. The method of expression and purification of the target viral peptides without or with the Trx tag is shown in Fig. 1B. Trx is an intracellular thermostable protein of *E. coli* that is highly soluble expressed in its cytoplasm^25^. It is one of the most frequently used fusion tags for enhancing the soluble expression of recombinant proteins in *E. coli* cells^25^,^26^. Herein, the Trx tag is incorporated to increase the solubility and stability of the target peptides after intein-mediated cleavage, particularly for peptides that are unstable in solution and more prone to form aggregates^27^,^28^,^29^. Here we discovered that in spite of its great solubility, the Trx tag was able to be first expressed as insoluble aggregates of fusion protein and subsequently released into the soluble fraction after intein-mediated cleavage, which was applicable with the cSAT scheme (Fig. 1B).

### Expression and purification of the target viral peptides using the cSAT scheme

The target viral peptides in this work included P1–P5 from the pandemic 2009 A(H1N1) influenza virus hemagglutinin (HA), and G9 and G31 from the HIV-1 envelop glycoprotein gp120, all of which were obtained by screening random viral peptide libraries in previous studies^23^,^24^. The lengths of the above viral peptides ranged from 32 to 72 aa, and the sequences are listed in Supplementary Table S1.

**Figure 2:**
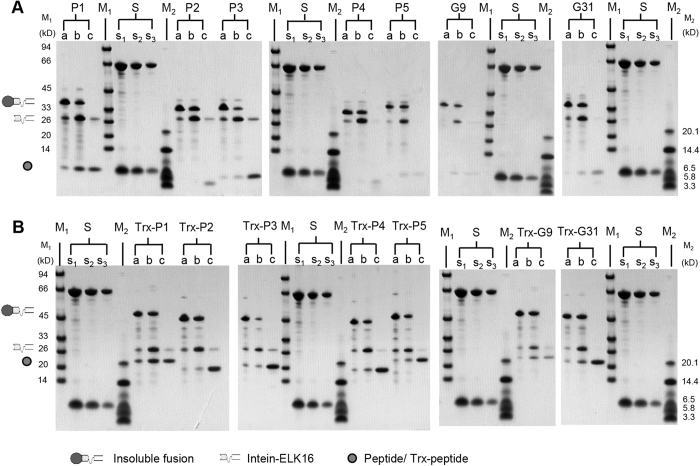
Expression and purification of target viral peptides. Expression and purification of the 2009 A (H1N1) influenza hemagglutinin (HA) peptides P1–P5 and HIV-1 (HXB2) envelope glycoprotein GP120 peptides G9, G31 without (**A**) and with (**B**) Trx tag, as detected by SDS-PAGE. Lane a, insoluble fraction of the cell lysate; lanes b and c, insoluble and soluble fractions of the cleaved fusion protein respectively. The loading volume of the protein samples was 1:2:4 for lanes a, b and c respectively. Lanes s1, s2 and s3, quantification standards consisting of bovine serum albumin (BSA, 66.5 kDa) at 3, 1.5 and 0.75 μg/lane and aprotinin (6.5 kDa) at 1.5, 0.75 and 0.3 μg/lane respectively. The molecular weights of the protein standards M1 and M2 are indicated on the left and right sides respectively. The gels were cropped and the full-length gels are presented in Supplementary Fig. S4.

The expression and intein-mediated cleavage results for the fusion of target peptide-intein-ELK16 are shown in Fig. 2A. All of the target peptides accumulated as insoluble aggregates at an estimated concentration of 15.0–31.3 μg/mg wet cell weight, based on the densitometry analysis of the SDS-PAGE results (Table 1). The aggregates were isolated by centrifugation and intein-mediated cleavage was induced with 40 mM DTT at 4°C overnight. Four of the target peptides, P1, P2, P3 and G31 were successfully released into the soluble fraction after cleavage, with yields estimated to be 1.0–3.6 μg/mg wet cell weight and recovery rates of 24.8–65.8%. The purities of these four peptides were estimated to be in the range of 62.1–69.9% (Table 1). However, three of the target peptides, P4, P5 and G9, were poorly released into the soluble fraction, with yields lower than 0.5 μg/mg wet cell weight, which were lower than the detection range.

The results obtained with the Trx-target peptide-intein-ELK16 fusion are shown in Fig. 2B. The fusion aggregates were expressed at an estimated level of 18.9–36.8 μg/mg wet cell weight (Table 1). All of the target peptides with the N-terminal Trx tag were successfully released into the soluble fraction after intein-mediated cleavage. With the exception of Trx-G9, all of the other peptides were produced in high yield, estimated to be around 4.8–7.1 μg/mg wet cell weight with recovery rates of 30.0–78.1%. As for Trx-G9, the majority of the target peptide remained in the insoluble fraction, with a soluble peptide yield of 0.9 μg/mg wet cell weight and a recovery rate of 8.3%. The purities of the target peptides containing the Trx tag that were released into the soluble fraction were also higher than those without the Trx tag, and were estimated to be 67.1–98.3% (Table 1).

### Antigenic activity assay of the purified peptides

**Figure 3:**
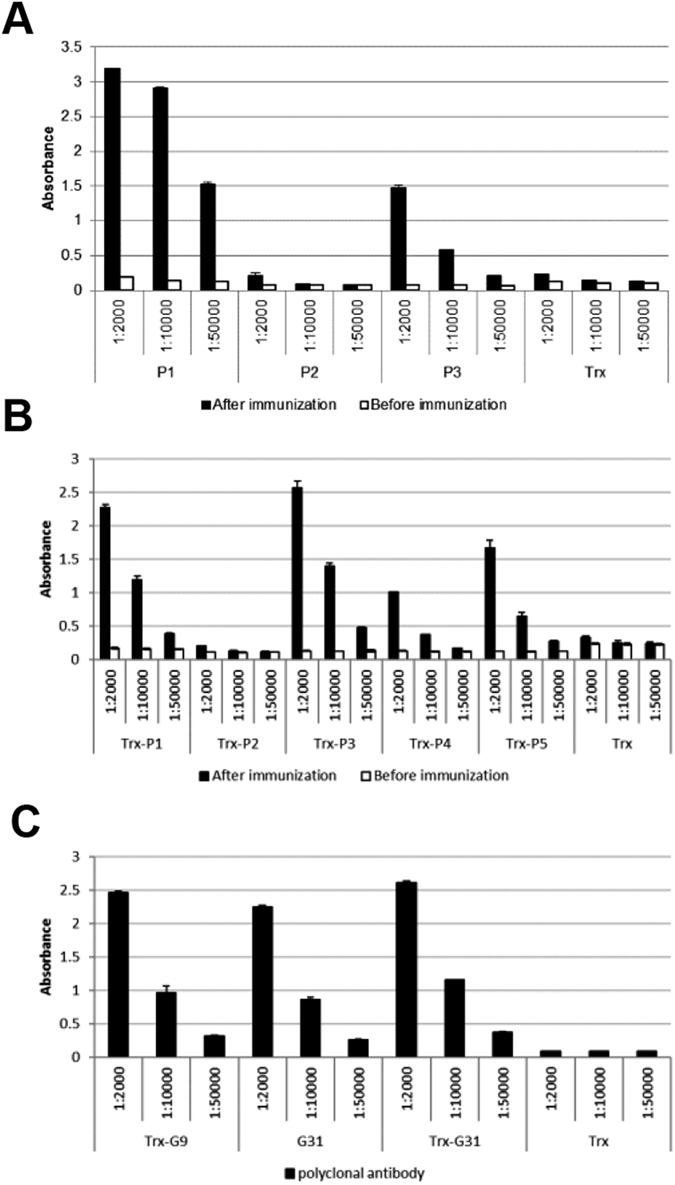
Antigenic activities of the target viral peptides as characterized by ELISA. Binding activities of the HA peptides P1–P3 (**A**) and P1–P5 with the N-terminal Trx tag (**B**) against the goat antisera before and after immunization, and GP120 peptides G9 and G31 without or with Trx tag against goat anti-GP120 polyclonal antibody (**C**), as determined by ELISA. Trx was used as the negative control. The x-axis shows the dilution ratios of the corresponding antisera or polyclonal antibody. The y-axis shows the absorbance at 450 nm after development with the substrate 3,3′,5,5′-tetramethylbenzidine (TMB). (**B**) was reproduced from ref. 24.

The antigenic activities of the target viral peptides released into the soluble fraction from the fusion aggregates were verified by ELISA. In a previous study, the HA peptides P1–P5 were screened from a random peptide library using three kinds of antisera from mouse, goat and human immunized against 2009 A(H1N1) influenza virus HA. To characterize the antigenic activities of P1–P5 to the three kinds of antisera, the five target peptides with an N-terminal Trx tag were expressed and purified from *E. coli* using the cSAT scheme^24^. Here we also tested the antigenic activities of P1–P3 without the additional Trx tag. As shown in Fig. 3A,B, the binding activities of P1–P3 alone to the antisera from goats agreed with those of P1–P3 in fusion with Trx. Specifically, P1 was reactive to both the goat and mouse antisera, while P3 was only reactive to goat antisera (see Supplementary Fig. S1). It should be noted that although the strength of the binding activity of Trx-P3 to goat antisera was similar to that of Trx-P1, the activity of P3 alone was weaker compared with P1. We hypothesized that since P3 is a short peptide of only 39 aa, its antigenic structure may be stabilized by the presence of the Trx tag. Again, P2 showed no measurable binding activity to either of the antisera, probably because of its incorrect conformation as previously reported^24^. It is noteworthy that both Trx-P4 and Trx-P5 yielded detectable binding against goat antisera, whereas in the absence of the N-terminal Trx tag, P4 and P5 could not be obtained in sufficient amounts for the assay (Fig. 3B). The antigenic activities of P1–P5 with the N-terminal Trx tag, and P1–P3 alone against the mouse antisera are also shown in Supplementary Fig. S1. It is clear that the binding activities of P1–P3 to the mouse antisera agreed with those of P1–P3 fused with the Trx tag.

The peptides G9 and G31 were identified by screening for foldable fragments of the HV-1 envelope glycoprotein gp120, but the antigenic activities of these peptides remained to be elucidated^23^. In this study, a commercial goat-derived polyclonal antibody against HIV-1 (clone HXB2) gp120 was used to detect their binding activities by ELISA (Fig. 3C). All of the peptide samples purified from *E. coli* using the cSAT scheme, including G31, Trx-G9 and Trx-G31, were detected by the polyclonal antibody. It was thus confirmed that these two target peptides retained strong antigenic activities during the expression and purification process.

## Discussion

In this work we reported the recombinant expression and purification of seven viral antigenic peptides of 32 to 72 aa in length based on the cSAT scheme. The target peptides with or without the N-terminal Trx tag were first expressed as insoluble aggregates, then released into the soluble fraction and easily purified after intein-mediated self-cleavage. The binding activities of the purified viral peptides to the antisera or polyclonal antibodies were verified by ELISA.

Comparing the two types of fusion constructs (with or without the N-terminal Trx tag), the former performed better because the yields and purities of all tested viral peptides were greatly improved, especially for P4, P5 and G9, the yields of which were undetectable (<0.5 μg/mg wet cell weight) without the Trx tag. In particular, P4 and P5 when fused to Trx were produced at high yields in the soluble fraction. G9, on the other hand, while the yield was improved, most of the target peptide in the Trx fusion form still remained in the insoluble fraction after intein cleavage. The grand average of hydropathicity (GRAVY) value was calculated using web.expasy.org/protparam (see Supplementary Table S1) for all seven peptides^30^. As indicated, G9 is a hydrophobic peptide with a positive GRAVY value, whereas the other six peptides are all hydrophilic peptides with negative GRAVY values^30^. Therefore, we speculate that the hydrophobicity of the target peptide may still greatly affect its solubility when fused to Trx. Trx has also been reported to enhance the cytoplasmic solubility of proteins with disulfide bonds by the virtue of its intrinsic oxido-reductase activity^27^. In this regard, G9, which has two disulfide bonds, may also benefit from fusion to Trx, albeit in a lesser degree compared with P5 (with one disulfide bond). The Trx-peptides showed antigenic activity towards specific antisera. This would be especially beneficial when a panel of antigenic peptides of reasonable purity (>70%) are required quickly for parallel analysis of their activities using typical immunoassays, such as ELISA and immunoblotting^24^. The major impurity in the purified samples was the cleaved fusion tag intein-ELK16, which would not be expected to interfere with the assays in most cases. It should be mentioned that other solubility tags could also be incorporated into the cSAT scheme to assist with releasing the peptides into the soluble fraction.

As for the constructs without the Trx tag, the expression and purification results varied for different peptides. For P4, P5, and G9, the yields were low. We found that the cleavage efficiencies for P4, P5 and G9 fusion proteins were comparable with those for P1, P2 and G31 fusion proteins (Table 1), and for P3 fusion protein, it was even slightly higher. However, the fusion aggregate yields of P4, P5 and G9 were lower compared with the other peptides (Table 1). Moreover, the majority of these three peptides are distributed in the insoluble fraction after intein cleavage. This suggests that for these three peptides, the relatively stronger hydrophobicity or weaker hydrophilicity (see Supplementary Table S1), and the difficulty of forming correct double disulfide bonds during expression for P5 (1 disulfide bond) and G9 (2 disulfide bonds), affected the expression levels, as well as the solubility after intein-mediated cleavage. However, the scheme still provides a quick and low-cost method for producing peptides that can retain a stable conformation in the soluble fraction, as shown for P1–P3 from influenza HA and G31 from HIV-1 gp120.

The peptides produced in this study may then be applied in research focusing on the antigenic structure and vaccination potential of influenza and HIV viruses. For example, the P1 peptide correlated well with the epitope of a recently reported cross-neutralizing antibody 12D1 to several subtypes of influenza viruses^31^. Vaccination in mice using a chemical synthetic peptide mimicking the 12D1 epitope has been proven to provide protection against influenza viruses of the H3N2, H5N1 and H1N1 subtypes, and this 55-aa peptide was covered by the recombinant P1 peptide reported in the current study^32^. The G31 peptide corresponded to the conserved immunodominant C-terminal region of the HIV-1 envelop protein gp120. A chemical synthetic peptide of 27 aa, which was contained in the G31 peptide sequence, has been used as an antigen in a combined assay for HIV-1/HIV-2 infection^33^. Moreover, vaccination in mice with phage displaying the C-terminal region has been shown to induce neutralizing antibodies to HIV-1 clade C viruses^34^.

The increasing demand for antigenic peptides with good specificity and sensitivity requires more reliable, and preferably high-throughput, peptide synthesis techniques. The cSAT scheme described here provides a fast and efficient way to express and purify medium to large-sized recombinant peptides from *E. coli* cells, which can then be used in immunological assays to detect a broad range of viral antigens.

## Materials and Methods

### Materials

The DNA fragments for hemagglutinin (HA) peptides P1–P5 were amplified from plasmid CMV-R-Cali-04-09 carrying the whole HA gene of A/California/04/2009(H1N1) influenza virus, provided by Prof. Paul Zhou, Institute Pasteur of Shanghai, Chinese Academy of Sciences (Shanghai, China)^24^. The DNA fragments for GP120 peptides G9 and G31 were amplified from a synthetic GP120 sequence encoding HIV-1 (HXB2) envelop glycoprotein GP120^23^. The amino acid sequences of the above viral peptides are listed in Supplementary Table S1. Oligonucleotides for cloning were synthesized by Invitrogen (Shanghai, China). Restriction enzymes and DNA polymerases were purchased from New England Biolabs (Beverly, MA, USA) or Takara (Dalian, China). The vector pET30a and strain *E. coli* BL21(DE3) were from Novagen (Madison, WI, USA). The kits for DNA purification, gel recovery, and plasmid minipreps were obtained from Tiangen (Beijing, China). Sequencing was performed by Invitrogen or SinoGenoMax (Beijing, China). Mouse antisera, kindly donated by Dr. Zhonghua Liu, AIDS Research Center, School of Medicine, Tsinghua University (Beijing, China), were obtained by immunizing mice with the recombinant HA protein of A/California/04/2009(H1N1) virus. Hyperimmune goat sera, a gift from Dr. Guoyang Liao, Institute of Medical Biology of Chinese Academy of Medical Sciences and Peking Union Medical College (Kunming, China), were raised against a Chinese pandemic strain of 2009 A (H1N1) influenza virus. Goat anti HIV-1 GP120 polyclonal antibody was purchased from Meridian Life Science (Saco, ME, USA). Horseradish peroxidase (HRP)-labeled secondary antibodies were from Santa Cruz Biotechnology (Santa Cruz, CA, USA). All other chemicals were of analytical grade.

### Construction of expression vectors

Expression vectors pET-Target peptide (P)-Intein-ELK16(a) and pET-Trx-Target peptide (P)-Intein-ELK16(a) were based on plasmid pET30a-LipA-I-ELK16, constructed previously in our laboratory^22^. The *trx*A gene (GenBank: AAA24534.1) encoding thioredoxin (Trx) was amplified from the *E. coli* BL21 (DE3) genome using the primers Trx-For and Trx-Rev as listed in Supplementary Table S2, digested with *Nde*I and *Spe*I restriction enzymes, and then ligated with the similarly digested pET30a-LipA-I-ELK16. The resultant plasmid pET30a-Trx-I-ELK16 contained two additional *Bgl*II and *Eco*RV sites in between the Trx and intein sequences. For the expression vector pET-P-Intein-ELK16(a), the respective target peptide sequence was inserted into the *Nde*I and *Eco*RV sites of pET30a-Trx-I-ELK16. For the expression vector pET-Trx-P-Intein-ELK16(a), the sequence of G31, one of the target peptides, was amplified with the introduction of a GS linker sequence followed by a *Bgl*II site in the 5′-terminus using the primers G31-GS Linker-For and G31-Rev as listed in Supplementary Table S2. It was then digested with the *Bam*HI and *Eco*RV restriction enzymes and inserted into the *Bgl*II and *Eco*RV sites of pET30a-Trx-I-ELK16 with the same cohesive ends, yielding the expression vector pET-Trx-G31-Intein-ELK16(a). Then the other target peptide sequences could be directly inserted into the *Bgl*II and *Eco*RV sites of pET-Trx-G31-Intein-ELK16(a). *E. coli* BL21 (DE3) cells were used throughout for cloning and protein expression.

### Protein expression

*E. coli* BL21(DE3) cells harboring pET-P-Intein-ELK16(a) or pET-Trx-P-Intein-ELK16(a) corresponding to the respective target peptides were inoculated into Luria-Bertani (LB) medium supplemented with 50 mg/L kanamycin and incubated at 37 °C with shaking (250 rpm). Isopropyl β-D-1-thiogalactopyranoside (IPTG) was added to a final concentration of 0.2 mM to initiate protein expression when the OD_600_ reached 0.4–0.6. Culturing was continued for an additional 10 h. For expression temperatures, 23 °C, 30 °C, 37 °C were tested, with G31 (and Trx-G31) used as the model peptide. As shown in Supplementary Fig. S2, the amounts of aggregates produced at the three temperatures were similar, however, the peptide yield was lesser at the two higher temperatures. For the present study, we thus used the temperature 23 °C. Then cells were harvested by centrifugation at 8000 rpm for 20 min and the pellets were stored at −70 °C until further analysis.

### Peptide purification by intein-mediated cleavage and quantification

Harvested cell pellets were re-suspended in buffer B1 (20 mM Tris-HCL, 500 mM NaCl, 1 mM EDTA, pH 8.5) to 10 OD culture/ml, followed by sonication (Ultrasonic crasher, Scientz JY92-IIN, Ningbo, China). The soluble fractions were isolated from the lysates by centrifugation at 11,000 rpm for 10 min at 4 °C. The precipitates were washed twice with buffer B1, resuspended in the same volume of Buffer B3 (20 mM Tris-HCl, 500 mM NaCl, 1 mM EDTA, 40 mM Dithiothreitol, pH 8.5). Intein-mediated cleavage reactions were performed by incubating the samples at 4 °C overnight. Then the soluble and insoluble fractions were separated by centrifugation at 11,000 rpm for 15 min at 4 °C. The protein samples were analyzed by denaturing polyacrylamide gel electrophoresis using precast NuPAGE^®^ precast 4–12% Bis-Tris gels from Invitrogen, followed by staining with Coomassie Brilliant Blue G-250. The compositions and protein concentrations of all samples were determined densitometrically with Quantity One software (Bio-Rad Laboratories, Hercules, CA, USA) using bovine serum albumin (BSA) and aprotinin as standards, and were adjusted according to the loading volume of the protein samples.

### ELISA assay for purified peptides with or without the Trx tag

The soluble fractions containing HA and GP120 peptides with or without the Trx tag released after intein-mediated cleavage were desalted and concentrated by ultrafiltration with an Amicon^®^ Ultra-4 centrifugal filter device (3,000 molecular weight cutoff). The concentrations of the peptide samples were further determined colorimetrically using the Pierce^®^ Bicinchoninic Acid (BCA) Protein Assay Kit from Thermo Scientific (Rockford, IL, USA). For comparison, Trx purified from *E. coli* following the same procedure was used as the negative control. Next, 96-well plates were coated with 100 ng of purified peptide or Trx in 100 μL of sodium carbonate bicarbonate buffer (0.5 M, pH 9.6) at 4 °C overnight. After blocking with 10% fetal bovine serum diluted in 1× phosphate buffered saline (PBS) with 0.25% Tween-20 (PBST), serial dilutions of the respective antisera or polyclonal antibody were added to each well and incubated for 1 h at 37 °C, followed by the addition of 1:5000 diluted HRP-conjugated secondary antibody. Assays were developed by adding 100 μL of 3,3′,5,5′-tetramethylbenzidine (TMB) substrate solution and the reactions were stopped with 50 μL of H_2_SO_4_ (1 M). The assays were carried out in triplicate and each well was washed four times with PBST between steps. The absorbance at 450 nm was recorded on a SpectroMAX 190 Microtiter reader (Molecular Devices, Sunnyvale, CA, USA).

## Additional Information

**How to cite this article**: Xu, W. *et al.* Recombinant production of influenza hemagglutinin and HIV-1 GP120 antigenic peptides using a cleavable self-aggregating tag. *Sci. Rep.* **6**, 35430; doi: 10.1038/srep35430 (2016).

**Publisher’s note**: Springer Nature remains neutral with regard to jurisdictional claims in published maps and institutional affiliations.

## Supplementary Material

Supplementary Information

## Figures and Tables

**Table 1:** Quantification of peptides alone or fused with the Trx tag.

Target peptide	MW (kD)	Aggregate yield^a^ (μg/mg wet cell pellet)	Peptide yield^b^ (μg/mg wet cell pellet)	Cleavage efficiency^c^	Percent recovery^d^	Peptide purity
Peptide						
P1	8.4	31.3	3.6	69.1	43.1	67.4
P2	4.2	25.8	1.0	53.6	24.8	62.1
P3	4.3	27.8	3.0	82.3	65.8	69.9
P4	3.6	20.4	ND^e^	64.8	ND^e^	ND^e^
P5	7.1	15.1	ND^e^	53.5	ND^e^	ND^e^
G9	7.7	15.0	ND^e^	61.4	ND^e^	ND^e^
G31	6.2	22.1	1.5	52.8	31.1	66.0
Peptide with Trx tag						
Trx-P1	21.6	36.8	5.1	68.9	30.0	81.6
Trx-P2	17.4	31.6	6.3	62.9	48.9	87.6
Trx-P3	17.5	18.9	6.1	87.8	78.1	84.3
Trx-P4	16.8	22.7	5.9	69.4	53.0	93.9
Trx-P5	20.3	24.6	4.8	71.8	48.6	92.3
Trx-G9	20.9	23.7	0.9	52.9	8.3	67.1
Trx-G31	19.4	36.2	7.1	63.2	44.8	98.3

^a^Yield of protein aggregate and

^b^yield of free target peptide released after cleavage from LB culture with a wet cell weight of 2.66 ± 0.99 mg/ml.

^c^Cleavage efficiency was calculated by dividing the amount of cleaved protein aggregate by that of the total aggregate before cleavage.

^d^Percent recovery in terms of mass was calculated by dividing the mass of the free peptide released after cleavage by that that could be theoretically obtained from the respective protein aggregate, assuming complete cleavage and release.

^e^Out of the detection range.
