# Supplementary material for: Recombinant production of influenza hemagglutinin and HIV-1 GP120 antigenic peptides using a cleavable self-aggregating tag
Source: Sci Rep. 2016 Nov 3;6:35430. doi: 10.1038/srep35430 (PMC5093863; doi:10.1038/srep35430)
Supplement: Supplementary Information [file srep35430-s1.pdf]

Supplementary Information

Recombinant production of influenza hemagglutinin and HIV-1 GP120 antigenic peptides using a cleavable self-aggregating tag

Wanghui Xu[1,2], Qing Zhao[1], Lei Xing[1,3] & Zhanglin Lin[1,*]

[1] *Department of Chemical Engineering, Tsinghua University, One Tsinghua Garden Road, Beijing 100084, China*

[2] *Current address: Novozymes, China Headquarters, 14 Xinxi Road, Shangdi Zone, Haidian District, 100085 Beijing, China*

[3] *Current address: China National Petroleum & Chemical Planning Institute. 16th Floor, 7 Block, Hepingli Zone, 100013, Beijing, China*

* *Corresponding author: zhanglinlin@mail.tsinghua.edu.cn*

Peptide	Residue number	Position[a]	Sequence	GRAVY[b]
**2009 A (H1N1) influenza virus hemagglutinin peptides**				
**P1**	70	423-492	EKRIENLNKKVDDGFLDIWTYNAELLVLLENERTLDYHDSNVKNLYEKVRSQLKNNAKEIGNGCFEFYHK	-0.899
**P2**	37	387-423	LKSTQNAIDEITNKVNSVIEKMNTQFTAVGKEFNHLE	-0.562
**P3**	39	22-60	IGYHANNSTDTVDTVLEKNVTVTHSVNLLEDKHNGKLCK	-0.574
**P4**	32	308-339	PFQNIHPITIGKCPKYVKSTKLRLATGLRNIP	-0.281
**P5**	65	61-125	LRGVAPLHLGKCNIAGWILGNPECESLSTASSWSYIVETPSSDNGTCYPGDFIDYEELREQLSSV	-0.192
**HIV-1 (HXB2) gp120 peptides**				
**G9**	72	207-278	KVSFEPIPIHYCAPAGFAILKCNNKTFNGTGPCTNVSTVQCTHGIRPVVSTQLLLNGSLAEEEVVIRSVNFT	0.197
**G31**	48	431-478	SNNESEIFRPGGGDMRDNWRSELYKYKVVKIEPLGVAPTKAKRRVVQR	-1.102

Supplementary Table S1. Peptide sequences. [a] amino acid positions of the peptides on the respective intact viral envelope protein.[b] GRAVY: Grand average of hydropathicity, The GRAVY value for a peptide or protein is calculated as the sum of hydropathy values of all the amino acids, divided by the number of residues in the sequence.GRAVY value indicates the hydropathy of proteins: a positiveGRAVY score means the protein is hydrophobic anda negative score hydrophilic[1].

Primer name	Nucleotide sequence[a]	Description
**Trx-For**	5'-AGTTACATATGAGCGATAAAATTATTC-3'(*Nde*I)	Used to amplify the *trx*A gene, with the introduction of *Bgl* II and *Spe* I sites in the 3'- terminus, and insert between the *Nde* I and *Spe* I sites of pET30a-LipA-I-ELK[L. Xing, MCF, 2011].
**Trx-Rev**	5'-TCACGACTAGTGCATCTCCCGTGATGCACATTCGCATGATATCTGAACCAGATCTCGCCAGGTTAGCGTCGAGGAAC-3' (*Spe* I, *Eco*R V, *Bgl* II)	
**G31-GS linker-For**	5'-ATCGTGGATCCGGTTCAGGTTCAGGTTCTAGATCTTACAGCAACAACGAGTCTGA-3'(*Bam*H I, *Bgl* II)	Use to amplify the G31 gene, with the introduction of GS linker sequence in the 5'- terminus, and insert between the *Bgl* II and *Eco*RV sites of pET30a-Trx-I-ELK.
**G31-Rev**	5'-AGTTCGATATCACGTTGAACAACACGGCGC-3' (*Eco*R V)	

Supplementary Table S2. Oligonucleotides used for the construction of expression vectors.[a]The underlined nucleotides indicate restriction sites in the brackets.

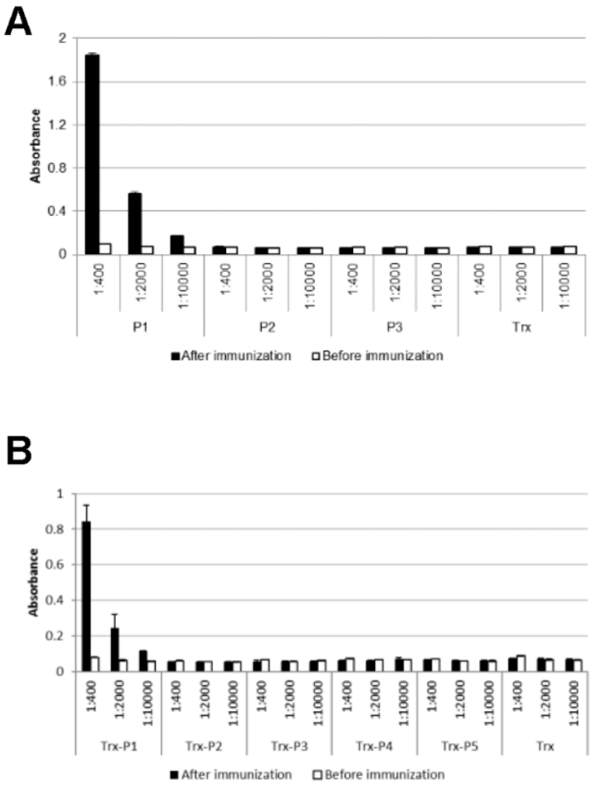

Supplementary Figure S1. Antigenic activities of P1–P5 with or without the Trx tagto mouse antisera

Binding activities of HA peptides P1–P3 (**A**) and P1–P5 with the N-terminal Trx tag (**B**) against the mouse antisera before and after immunization. Figure S1(B) was reproduced from reference 24[2].

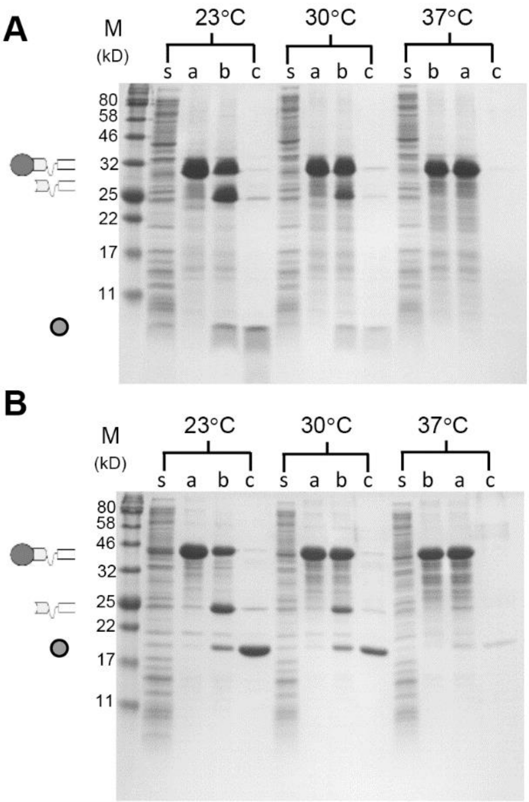

Supplementary Figure S2. Expression temperature optimization.

Expression and purification of the HIV-1 (HXB2) envelope glycoprotein GP120 peptides G31 without (**A**) and with (**B**) Trx tag at three different expression temperatures, as detected by SDS-PAGE. Lane s, soluble fraction of the cell lysate; Lane a, insoluble fraction of the cell lysate; lanes b and c, insoluble and soluble fractions of the cleaved fusion protein respectively.

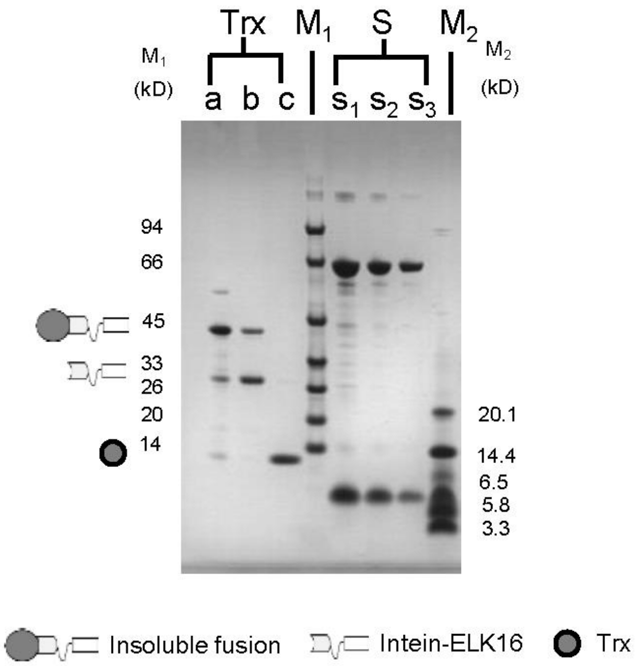

Supplementary Figure S3. The full-length gel of the gel in Figure 1.

SDS-PAGE results of Trx. Lane a, insoluble fraction of the cell lysate; lanes b and c, insoluble and soluble fractions of the cleaved fusion protein respectively; lanes s1, s2 and s3, quantification standards consisting of bovine serum albumin (BSA, 66.5 kDa) at 3, 1.5 and 0.75 μg/lane and aprotinin (6.5 kDa) at 1.5, 0.75 and 0.3 μg/lane respectively. The molecular weights of the protein standards M1 and M2 are indicated on the left and right sides respectively.

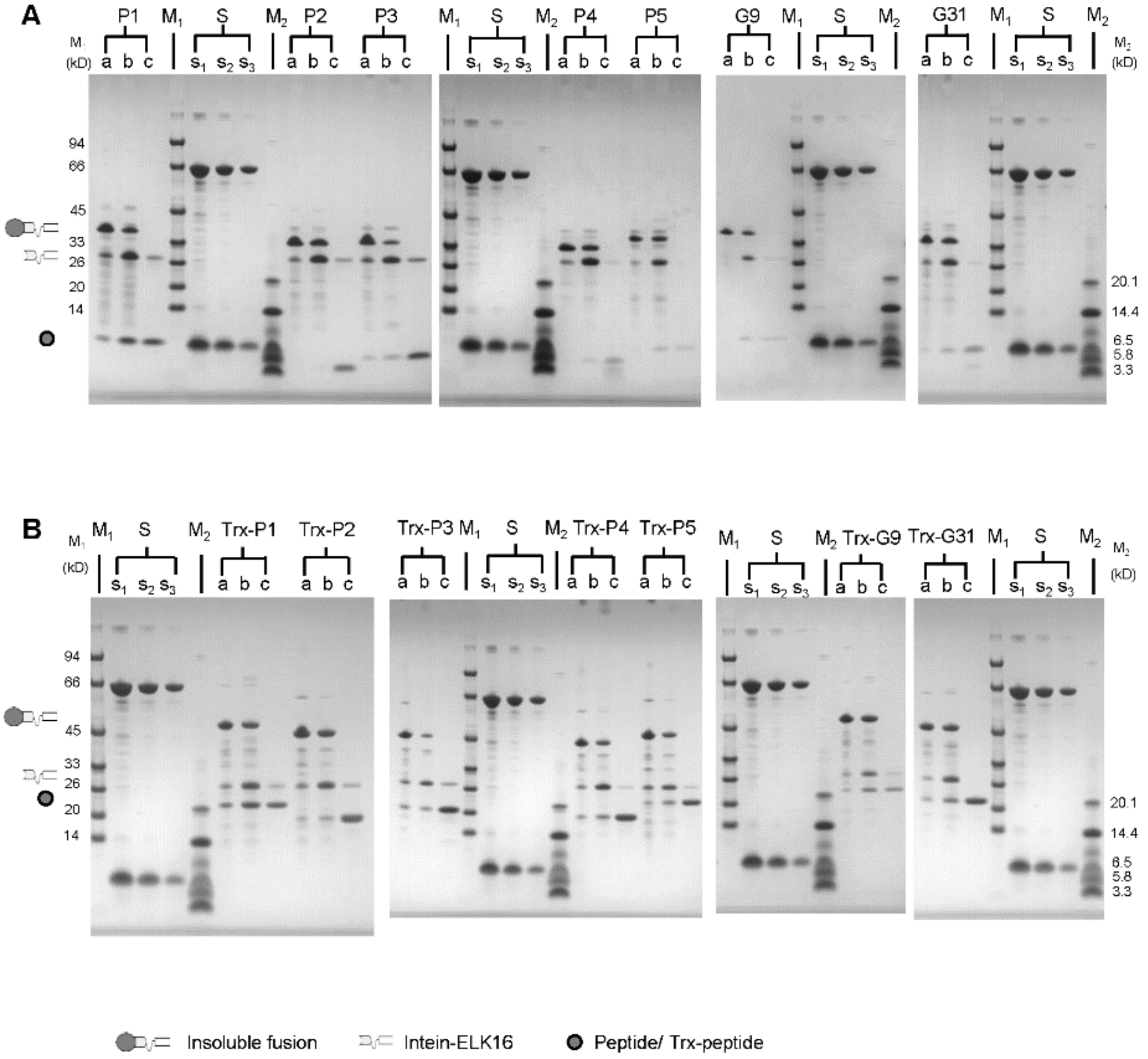

Supplementary Figure S4. The full-length gel of the gel in Figure 2.

Expression and purification of the 2009 A (H1N1) influenza hemagglutinin (HA) peptides P1–P5 and HIV-1 (HXB2) envelope glycoprotein GP120 peptides G9, G31 without (A) and with (B) Trx tag, as detected by SDS-PAGE. Lane a, insoluble fraction of the cell lysate; lanes b and c, insoluble and soluble fractions of the cleaved fusion protein respectively. The loading volume of the protein samples was 1:2:4 for lanes a, b and c respectively. Lanes s1, s2 and s3, quantification standards consisting of bovine serum albumin (BSA, 66.5 kDa) at 3, 1.5 and 0.75 μg/lane and aprotinin (6.5 kDa) at 1.5, 0.75 and 0.3 μg/lane respectively. The molecular weights of the protein standards M1 and M2 are indicated on the left and right sides respectively.

References

1. Kyte, J. & Doolittle, R. F. A simple method for displaying the hydropathic character of a protein. *J Mol Biol* **157**, 105-132 (1982).
2. Xu, W., Han, L. & Lin, Z. Screening of random peptide library of hemagglutinin from pandemic 2009 A(H1N1) influenza virus reveals unexpected antigenically important regions. *PloS one* **6**, 1-11 (2011).
